# Subversion of infiltrating prostate macrophages to a mixed immunosuppressive tumor‐associated macrophage phenotype

**DOI:** 10.1002/ctm2.581

**Published:** 2022-01-24

**Authors:** Clovis Boibessot, Oscar Molina, Gabriel Lachance, Christophe Tav, Audrey Champagne, Bertrand Neveu, Jean‐François Pelletier, Frédéric Pouliot, Vincent Fradet, Steve Bilodeau, Yves Fradet, Alain Bergeron, Paul Toren

**Affiliations:** ^1^ Centre de recherche du CHU de Québec—Université Laval Axe Oncologie Québec Canada; ^2^ Centre de recherche sur le cancer de l'Université Laval Québec Canada; ^3^ Département de chirurgie Université Laval Québec Canada; ^4^ Centre de Recherche en Données Massives de l'Université Laval Québec Canada; ^5^ Département de biologie moléculaire biochimie médicale et pathologie Faculté de Médecine Université Laval Québec Canada

**Keywords:** CD163, immunosuppression, macrophages, phenotype, prostate cancer

## Abstract

Tumor‐associated macrophages (TAMs) support tumor progression within the tumor microenvironment (TME). Many questions remain as to the origin, development, and function of TAMs within the prostate TME. Evaluation of TAMs in prostate cancer (PCa) patients identified the immunosuppressive TAM marker CD163 in adjacent normal epithelium as an independent predictor of metastases or PCa death. Flow cytometry analyses identified prostate TAMs as frequently expressing both proinflammatory M1 (CCR7+) and immunosuppressive M2 (CD163+) markers. In vitro, we demonstrate PCa cells similarly subvert human M1 macrophages toward a mixed M1/M2 macrophage phenotype favoring tumor growth. Further the cytokine milieu‐induced transition between immunosuppressive M2 to proinflammatory M1 (M2→M1) macrophages is abrogated by the presence of PCa cells. RNA sequencing suggests alterations in chemokine expression in prostate TAMs due to the presence of PCa cells. Together, our results suggest that prostate TAMs originate from inflammatory infiltrating macrophages, which are then reprogrammed mainly by PCa cells, but also the cytokine milieu. A better understanding of this subversion of macrophages within the prostate may lead to novel treatment strategies.

## Graphical abstract

Our results demonstrate that mixed inflammatory and immunosuppressive macrophages found in the prostate develop because of prostate cancer contact. The presence of these macrophages in adjacent normal epithelium portends worse long‐term clinical outcomes for prostate cancer patients, highlighting the importance to develop effective strategies against their development.

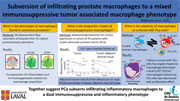

## 1 INTRODUCTION

Macrophages are known to quickly detect and adapt to changes in their microenvironment.^1^ They participate in the elimination of invading bodies or cell debris and drive inflammation to promote the recruitment of other immune cells and present antigens to T lymphocytes, therefore contributing to the shaping of the microenvironment.^2^ Tumor‐associated macrophages (TAMs) are a major component of the tumor microenvironment (TME) and play an important role in the progression of many cancers.^3^, ^4^ In contrast to normal macrophages, TAMs favor local immunosuppression, have lower cytotoxic function, decreased antigen presenting capability and promote matrix remodeling and angiogenesis.^1^, ^5^, ^6^, ^7^ However, the origin of TAMs is complex as they may originate from resident tissue macrophages or circulating bone marrow‐derived monocytes.^8^, ^9^, ^10^ Further understanding the plasticity of prostate cancer (PCa) TAMs is needed to aid the development of strategies to reprogram them towards an antitumoral phenotype.

Historically, macrophages were divided into classically activated M1 macrophages and alternatively activated M2 macrophages characterized by antitumor and protumor properties, respectively.^11^ However, this oversimplified binary classification is now referred to as a polarization spectrum to reflect the complexity and plasticity of macrophages within different contexts, including the TME.^12^ A recent study using single‐cell RNA sequencing in breast cancer samples reported expression of both M1 and M2 gene signatures in tumors.^13^ These findings were also reported in gliomas, suggesting a model wherein TAMs reside along a spectrum and not in mutually exclusive M1 or M2 polarization states.^14^

In prostate tumors, the TME is preferentially enriched with myeloid cells compared to lymphocytes in both human and murine models.^6^, ^15^, ^16^ TAMs, which can represent up to 30% of total tumor infiltrating immune cells, are characterized by an immunosuppressive phenotype.^17^ In general, a higher density of macrophages in the prostate is associated with a poorer prognosis,^18^ but the impact of macrophage characteristics on specific clinical events is not well understood. Prior research suggests that in localized tumors the expression of CD163 and CD206, both markers of M2 macrophages, is associated with an increased risk of developing metastases.^15^, ^19^, ^20^ Similarly, the proportion of CD206^+^ macrophages is increased in metastatic castration resistant prostate cancer (CRPC) compared to localized PCa.^21^ A recent analysis of PCa metastases suggest there exists two subtypes of CRPC: one characterized by higher androgen receptor (AR) and metabolic activities and a second characterized by high immune cell infiltration.^15^ Importantly, a meta‐analysis suggests that a higher density of prostate TAMs in localized disease is ultimately associated with poorer overall survival.^21^ Together, these suggest that the immune phenotype in early stage PCa may ultimately impact patient outcomes. With PCa poorly responsive to immune checkpoint‐targeted immunotherapies, a more detailed understanding of how macrophage biology in PCa could be important for the elaboration of novel therapeutic approaches.

**HIGHLIGHTS**

1. High expression of immunosuppressive macrophage marker CD163 in tumor‐adjacent normal epithelium independently predicts long‐term metastases or prostate cancer (PCa) death.
2. Direct contact with PCa cells strongly induces macrophage immunosuppressive markers.
3. Dual CCR7^+^/CD163^+^ macrophages induced by PCa cells lose PCa anti‐proliferative influence.
4. Mixed inflammatory and immunosuppressive prostate macrophages are common in men with Gleason grade group ≥3 localized prostate cancer.

In this study, we characterized TAMs infiltrating human PCa specimens and investigated the crosstalk between PCa cells and macrophages. Using human derived in vitro prostate TAM models, which reflect patient tumor findings, our analyses demonstrated the dual inflammatory and immunosuppressive nature of human prostate TAMs and the importance of a transition via the inflammatory M1 state to acquire this phenotype. Further, we identified CD163^+^ macrophages in normal appearing prostate tissue as prognostically important. Together, these data suggest that most TAMs in the PCa microenvironment are subverted inflammatory infiltrating macrophages. The resistance of these subverted macrophages to repolarize in the presence of PCa cells has implications for the development of effective immunotherapy against PCa.

## 2 MATERIALS AND METHODS

### 2.1 Patient samples

Informed, written consent was obtained from all participants. A clinical database recorded patient demographics, tumor clinicopathological characteristics, as well as data on biochemical recurrence, metastases, and death. The research ethics committee of the Centre Hospitalier Universitaire de Québec‐Université Laval (CHUQc‐UL) approved the use of patient specimens and clinical data for each cohort and male volunteers (#2012–1002; #2012–1059; #2019–4181). The first cohort consisted of 95 men with localized PCa treated by radical prostatectomy between 1996 and 1998. Formalin‐fixed paraffin‐embedded (FFPE) tumors from these patients were obtained and whole tumor sections were used for immunohistochemical analysis. A second cohort consisted of 21 men with Gleason Grade Group (GGG) ≥ 3 PCa on preoperative biopsy that underwent radical prostatectomy in 2019 or 2020.

### 2.2 Immunohistochemistry

FFPE prostate tumors were cut into 5‐μm‐thick sections and dried overnight at 37°C. Sections were deparaffinized, and heat‐induced antigen retrieval was performed using a PT Link, Pre‐Treatment Module for Tissue Specimens (Dako, Burlington, ON, Canada) with Tris/EDTA, pH 9 (Dako Code K8004: EnVision™ FLEX, High pH). Endogenous peroxidase activity was blocked by incubation in 3% peroxide solution for 10 min. The immunodetection was performed using the IDetect super stain HRP polymer kit (ID labs, London, Ontario, Canada) as follows. First, slides were incubated for 10 min at room temperature with Super block solution to block nonspecific background staining. Then, incubation with anti‐CD163 monoclonal antibody (mAb, clone 2G12, dilution 1:2000, Abcam, Toronto, ON) was carried out for 1 h at room temperature. After washes, slides were incubated for 30 min with HRP Polymer Conjugate according to manufacturer's recommendations. After a 5 min DAB staining, slides were rinsed, counterstained with hematoxylin, dehydrated and mounted with coverslip using MM 24 low viscosity mounting medium (Leica Microsystems, Durham, USA). Slides were digitalized using a Nanozoomer (Hamamatsu Photonics, Bidgewater NJ, USA) and visualized using the NDP.view2 software (Hamamatsu Photonics). The density of CD163^+^ cell infiltration was analyzed in tumor and normal‐appearing adjacent tissue. In each area, ten randomly selected visual fields at 20× magnification (surface area of 0.460 μm^2^) were chosen and the number of positive cells in these fields was determined by semiautomatic digitized image analysis using the Calopix software (RTIBVN Healthcare, Châtillon, France). For quality control, 10% of the slides were randomly selected and the scoring was confirmed by a genitourinary pathologist.

### 2.3 Cell culture

LNCaP cells were cultured in RPMI 1640 supplemented with 10% heat‐inactivated fetal bovine serum (FBS) (Wisent Bioproducts, St‐Bruno, QC, Canada). Enzalutamide‐resistant 49C^ENZR^ and 49F^ENZR^ cells (kindly provided by Dr. Amina Zoubeidi at the Vancouver Prostate Centre) were cultured in RPMI 1640 supplemented with 10% heat‐inactivated FBS and with 5‐μM enzalutamide (MedChemExpress LLC, Monmouth Junction, NJ, USA, #HY‐70002). PC3 and LAPC4 cells were cultured in Dubelcco's modified Eagle's minimal essential medium (DMEM medium, 1 g glucose/L) supplemented with 10% heat inactivated FBS. RWPE‐1 and PZ‐HPV7 cells were cultured in keratinocyte serum‐free medium supplemented with prequalified human recombinant epidermal growth factor 1–53, bovine pituitary extract and 10 nM dihydrotestosterone. Authentication of LNCaP, PC3 and RWPE‐1 cells was performed using the GlobalFiler IQC PCR Amplification Kit (ThermoFisher Scientific Inc., Ottawa, ON, Canada).

### 2.4 Monocyte‐derived macrophages preparation and polarization

Monocyte‐derived macrophages (MDMs) were prepared from PBMCs of healthy male volunteers aged 40–70 years. Whole blood was collected in EDTA tubes and PBMCs were isolated by FicollÒ PAQUE Plus density gradient centrifugation (GE Healthcare Life Sciences, endotoxin tested, #17‐1440‐02). Monocytes were purified from PBMCs by magnetic‐activated cell sorting positive selection with CD14 microbeads kit (Miltenyi Biotec, Gaithersburg, MD, USA, #130‐050‐20) and LS Columns (Miltenyi Biotec, #130‐042‐401). Purified CD14^+^ monocytes were seeded in 12‐well plates (1 × 10^6^ cells/well) or in 24‐well plates (4 × 10^5^ cells/well) in RPMI supplemented with 10% heat‐inactivated FBS, 2.38 g/L of D‐glucose (AnalR, #10117), 2.50 g/L of HEPES (Sigma‐Aldrich, Oakville, ON, Canada), and 10 ng/ml recombinant human monocyte‐colony stimulating factor (M‐CSF, Peprotech, Cranbury, NJ, USA, #300‐25) for 5 days to differentiate the monocytes into M0 macrophages. On day 5, 50% of volume of fresh medium supplemented with M‐CSF (10 ng/ml) was added. On day 6, M0 macrophages were polarized into M1 or M2 macrophages with 20 ng/ml of IFN‐γ (Cedarlane, Burlington, ON, Canada, #CL‐101‐06) and 10 ng/ml of LPS (Sigma‐Aldrich) for M1 macrophages; to obtain M2 macrophages, 20 ng/ml of interleukin‐4 (IL‐4; Cedarlane, #CL‐101‐04) and 20 ng/ml of IL‐13 (Cedarlane, #CL‐101‐13) were added to the medium. Culture was then continued for 4 days. For MDMs reeducated by cancer cells, 5 × 10^5^ MDMs were polarized into M1 or M2 macrophages in 6‐well plates as described above. Then, after 24 h of polarization, 5 × 10^5^ PCa cells were added to the wells for an additional 4 days of coculture. For MDMs reeducated by conditioned media, 5 × 10^5^ MDM were polarized as above for 48 h in 6‐well plates. After 48 h of polarization, supernatant was collected, cells were washed two times with PBS and conditioned supernatant from M1 macrophages was added to M2 macrophages and supernatant from M2 macrophages was added to M1 macrophages for an additional 2–6 days.

### 2.5 Macrophage analysis from fresh prostate tissue specimens

Fresh prostate biopsies were obtained from men undergoing radical prostatectomy as previously described.^22^ Briefly, six 18‐gauge needle biopsies (2 from the tumor area and 4 from the adjacent nontumor area) were washed and cultured for 72 h in Advanced DMEM‐F12 media supplemented with 50 mg/L of antimicrobial agent Primocin (InvivoGen, San Diego, CA), 5 ml/L of Glutamax (ThermoFisher Scientific, #35050061), 1489 mg/L of HEPES added with 10% of patients’ serum (autologous). Biopsies were first washed twice with HBSS with Ca^2+^ Mg^2+^, then incubated overnight at 37°C with 5% CO_2_ with Type II collagenase (ThermoFisher Scientific, #17101015, final concentration of 300 U/ml) and 2 U/ml of DNase (Sigma #10104159001) in fresh medium without autologous serum. Next, dissociated biopsies were washed with HBSS with Ca^2+^ Mg^2+^ and incubated with 1 ml Accutase (Corning, #25‐058‐ci) for 20 min at 37°C. Dissociated cells were then collected, washed, stained, and filtered for flow cytometry analyses.

### 2.6 Cytokine analyses

The levels of cytokines in the supernatant of cultured macrophages were determined using the Bio‐Plex Pro Human Cytokine Th1/Th2 immunoassay (Bio‐Rad, Mississauga, ON, Canada, #M5000005L3), which measures GM‐CSF, IFN‐g, IL‐2, IL‐4, IL‐5, IL‐10, IL‐12(p70), IL‐13, and TNF‐a using the Luminex technology. Medium alone was used as blank and nontreated samples were compared with treated samples. Assays were run on a Bio‐Plex® 200 System and data analyzed using Bio‐Plex Manager™ Software 6.1 (Bio‐Rad).

### 2.7 Proliferation assays

cells were centrifuged, washed two times with PBS and then incubated with 10 μM CFSE/1 × 10^6^ cells/mL for 20 min in the dark at room temperature. Cells were washed two times with complete medium then added to macrophage cultures for 96 h. After the incubation period, cells were harvested, marked with V500‐labeled anti‐CD45 mAb (Table S1) according to manufacturer's recommendation and then analyzed by flow cytometry.

### 2.8 Cell staining and flow cytometry

For flow cytometry analyses, controls included compensation beads (BD CompBeads, BD Biosciences, San Jose, CA, USA, #552843) and Fluorescence Minus One (FMO) performed on fresh samples to identify gating boundaries. For in vitro PCa cells and MDM, cells were mechanically detached from the plate and washed twice with PBS. For these cells as well as for those obtained after biopsy dissociation, cells were incubated with Seroblock (Bio‐Rad, #BUF070B) for 5 min, and then with a cocktail of mAbs against CD11b, HLA‐DR, CCR7, CD163, CD206, PD‐L1, PD‐1, and B7‐H3 (Table S1). Cells were analyzed using a BD LSRFortessa cytometer (BD Biosciences) and data analysis was performed using FlowJo software (v10.5.2) (Treestar, Inc., Ashland, OR). Dead cells were excluded using morphology and doublets based on forward scatter‐A against forward scatter‐H gating, with optimization studies indicating approximately 10% of nonviable CD45^+^ cells were missed when compared with BD Horizon fixable viability stain (FVS‐780, BD Biosciences).

### 2.9 Fluorescent activating cell sorting

Macrophages were harvested and labeled with viability stain FVS‐780, V500‐labeled anti‐CD45, PE‐labeled anti‐CCR7 and AF‐647‐labeled anti‐CD163 mAbs and sorted on ARIA II flow cytometer (BD Biosciences). Sorted cells were maintained in complete medium supplemented with Primocin before being snap frozen for further RNA sequencing or seeded in 24‐well plate for 6 h before an additional 72 h of coculture with 49C^ENZR^ cells.

### 2.10 High‐dimensional visualization of flow cytometry data

For visualization of pooled flow cytometry results, a t‐SNE map was created using FlowJo software. The global geometry was evaluated with 3 different values of perplexity (30, 50, 100) and steps (1000, 3000, 5000), with values of 100 for perplexity and 5000 for geometry selected based on the maximum resolution for total immune cells. Major clusters were identified by manually gating in different populations by the fluorescence intensity of selected markers, with gates overlayed on the t‐SNE maps.

### 2.11 Gene expression profiling dataset

Clinicopathological and gene expression data from TCGA‐PRAD [18] and GSE21032 [19] were obtained from GDC (Genomic Data Common; https://portal.gdc.cancer.gov/) and GEO (Gene Expression Omnibus; https://www.ncbi.nlm.nih.gov/geo/) data portals (*n* = 333 and *n* = 218, respectively). The expression of *CCR7*, *CD163*, *CD276*, *CD274*, and *MRC1* in these mRNA datasets was analyzed using GraphPad Prism 8.0.

### 2.12 RNA‐sequencing

Details on RNA sequencing and the bioinformatics pipeline are provided in Supplementary Methods. Briefly, following library preparation sequencing was performed an NovaSeq 6000 flowcell S2 Illumina sequencer at the Genomics platform at the CHUQc‐UL Research Center with a mean coverage of ∼22 M paired‐end reads. Following bioinformatics processing, the 500 genes with the highest variance across samples were then analyzed using Principal Component Analysis (PCA) from the prcomp function in R (https://www.r‐project.org) and visualized using the plotly R package (https://plot.ly). Differentially expressed genes were identified with DESeq2^23^ (genes with zero coverage in all samples were excluded) and called with a significance at Benjamini–Hochberg corrected *p* < .05. Upregulated genes were selected at a minimum log2 fold change of 1.5 and downregulated genes at a minimum log2 fold change of –1.5.

### 2.13 Statistical analyses

For immunohistochemistry results, the CD163^+^ cell count was categorized into quartiles. Survival was compared with Kaplan–Meier curves and the log‐rank test. Univariate and multivariable proportional hazard Cox models assessed the effect of CD163 immune cell infiltration on clinical outcome (metastasis and PCa‐specific death), with propensity score adjustment using age, PSA, stage, Gleason score, and margin status. Time‐to‐event variables were calculated from the date of radical prostatectomy. Statistical analyses were performed using SAS Statistical Software v.9.4 (SAS Institute, Cary, NC, USA) with a two‐sided significance level set at *p* < .05. For in vitro and flow cytometry results, normality was evaluated by both D'Agostino & Pearson normality test and Shapiro–Wilk normality test using GraphPad Prism 8.0. For nonparametric distributions, unpaired Mann–Whitney test or paired Wilcoxon matched‐pairs signed rank test were used. For parametric distributions, Student's *t*‐test was used.

## 3 RESULTS

### 3.1 CD163^+^ macrophages in tumor‐adjacent normal areas predict clinical outcomes

**FIGURE 1:**
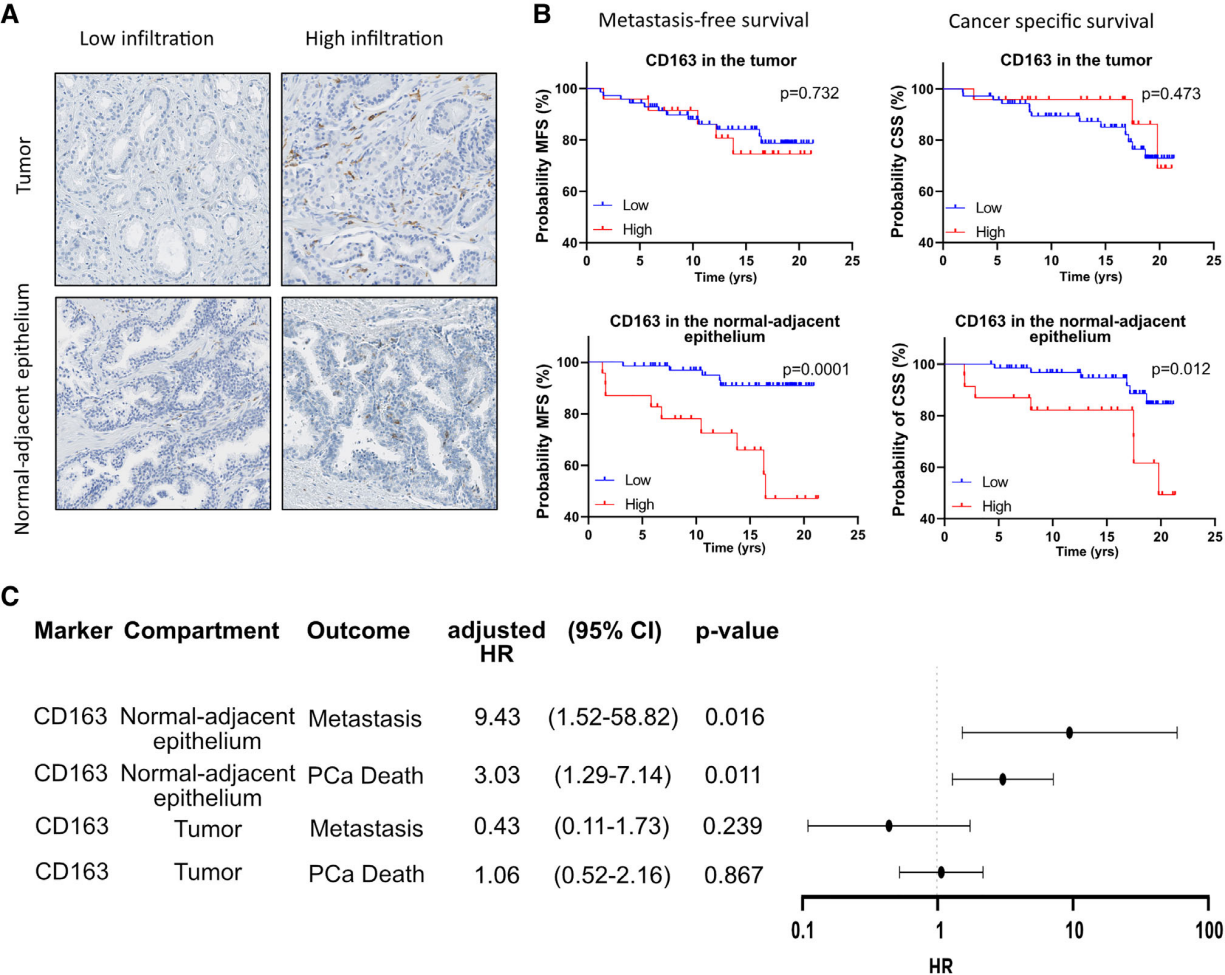
TAMs expressing CD163 within the surrounding tumor environment predict poor prognosis. Immunohistochemistry analysis of the prognostic significance of CD163 infiltration in 95 PCa samples. The levels of CD163+ cell infiltration in tumor and tumor‐adjacent normal epithelia were determined and data were categorized as quartiles. (A) Examples of low and high infiltration of CD163+ macrophages in each tumor area are provided. (B) The prognostic significance of CD163+ cell infiltration in tumor and tumor adjacent normal epithelia was analyzed by Kaplan–Meier and Cox regression analyses. Kaplan–Meier curves showing PCa metastasis‐free survival (MFS) and PCa‐specific survival (CSS) according to the level of CD163+ macrophage infiltration (4th quartile/high [red line] vs. 1st–3rd quartiles/low [blue line]) in the tumor and in the tumor‐adjacent normal epithelium areas are presented. Log‐rank test was used to assess the significance of the differences observed. (C) Multivariable Cox regression analyses also showed that infiltration of CD163+ macrophages in the tumor‐adjacent normal epithelia (CD163N), but not in the tumor (CD163T), is associated with a significant higher risk of metastasis and PCa specific death as shown in the forest plot. Multivariable analyses were adjusted for age, PSA, T stage, N Stage, Gleason, and surgical margin status

To understand the impact of immunosuppressive TAMs in the TME of PCa, we evaluated CD163 staining in a cohort of 95 locally advanced PCa radical prostatectomy specimens selected for adverse pathology (pT2 with positive margins or ≥pT3, Table S2). The median follow‐up of this cohort is 15.5 years. We first manually delineated the TME into tumor and tumor‐adjacent normal areas to evaluate the impact of localization on clinical outcomes (Figure S1A, B). CD163^+^ cells were scattered throughout the tissue and a wide intrapatient variation in staining was observed. However, the staining pattern was similar from one patient to another: positive cells were more abundant in the tumor areas (mean of 23 cells/visual field) than in the adjacent normal areas (mean of 14 cells/visual field, Figures 1A and S1A). Survival analysis demonstrated that a high CD163^+^ cell infiltration (4th quartile) in the tumor‐adjacent normal‐like epithelium, but not in the tumor, was significantly associated with shorter survival without metastases (*p* *= *.0001) and PCa‐specific mortality (*p* *= *.0124, Figure 1B) as well as with CRPC‐free survival (*p* *= *.016, Figure S1B). In multivariable analyses, a high CD163^+^ cell infiltration in tumor‐adjacent normal‐like epithelium significantly increased the risk of developing metastases (adjusted hazard ratio [HR] = 9.43, 95% confidence interval (CI) 1.52–58.82, *p* *= *.016) and PCa‐specific death (adjusted HR = 3.03, 95% CI 1.28–7.14, *p* *= *.011, Figure 1C) as well as CRPC (adjusted HR = 4.88, 95% CI 0.97–24.39, *p* *= *.05, Table S3). In contrast, CD163^+^ cell infiltration in tumor area was not associated with any of these outcomes (Figure 1C, Table S3). These findings demonstrate the prognostic importance of immunosuppressive macrophages in locally advanced prostate tumors, with intriguing findings that the level of infiltration of CD163^+^ macrophages in tumor‐adjacent normal‐like epithelium is the strongest independent predictor of adverse clinical outcomes.

### 3.2 Dual CD163^+^/CCR7^+^ macrophages are present in tumor‐adjacent normal‐like epithelium

To better understand macrophages within the prostate TME, we analyzed their phenotype in ex vivo cultured prostate biopsies using multiparametric flow cytometry. Only patients with Gleason Grade Group ≥ 3 were included to avoid indolent tumors. From each patient a pool of 6 prostate needle biopsies were evaluated using our optimized dissociation protocol.^22^ Since CD163^+^ macrophages in the tumor‐adjacent normal‐like epithelium were the strongest prognostic factor for outcomes above, 2 of these 6 biopsies were taken from the tumoral zones and 4 from the adjacent sextants based on preoperative biopsy parameters.

**FIGURE 2:**
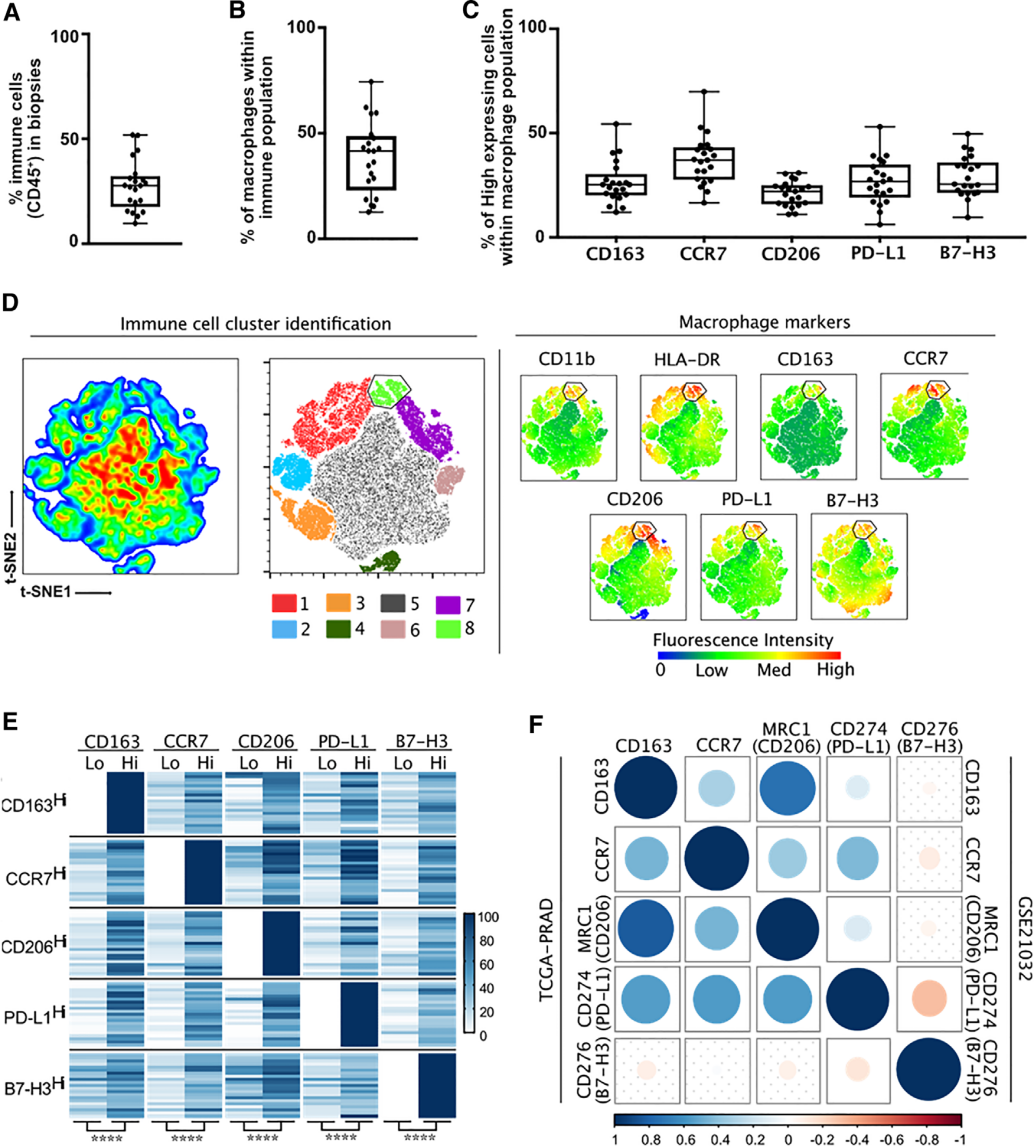
Prostate tumors and adjacent normal tissues contain macrophages expressing both M1 and M2 characteristics. Using patient cohort 2, a total of 6 needle biopsies (2 from the tumor and 4 from the adjacent normal areas) were taken in the prostate of patients (*n* = 18) with Gleason Grade Group ≥ 3 that underwent radical prostatectomy. Macrophage infiltration was evaluated by flow cytometry analysis using common macrophage markers (HLA‐DR, CD11b), M1 marker (CCR7), M2 markers (CD163, CD206), and immune checkpoints (PD‐L1 and B7‐H3). (A) Proportion of total immune cell infiltration (CD45+), (B) proportion of macrophages within immune population (HLA‐DR+ CD11b+), and (C) proportion of M1 (CCR7), M2 (CD163, CD206), or immune checkpoint (PD‐L1 and B7‐H3) high expression within the macrophage population are presented. Data from all tumors were pooled and major immune cell clusters (1) to (8) were evaluated by smooth density plots. Each color represents one immune cluster based on the differential expression of selected macrophages markers (left). (D) t‐SNE mapping was used to highlight the macrophage population within total immune cell population by coexpression of macrophages markers (right). (E) Heatmap shows the frequency of M1 and M2 markers high expression within the manually gated low and high expressing clusters from each marker in each tumor. Scale bar indicates frequency of high expression from 0% (white) to 100% (dark blue) of the indicated markers. High and low fractions were analyzed by Student's *t*‐test. ^****^ indicates *p* = .0001. (F) To corroborate data obtained using flow cytometry, coexpression of the genes corresponding to the macrophages markers was analyzed in two PCa RNA datasets. Spearman's correlation matrix of gene expression levels extracted from TCGA‐PRAD (bottom left) or GSE21032 (top right) is presented. The degree of negative correlation (red) and positive correlation (blue) is indicated by the color intensity and the size of the circles. Nonsignificant correlations are identified with dotted squares

In the dissociated biopsies from 21 patients, approximately 25–30% of the viable cells represented immune lineage cells (CD45^+^ cells, Figure 2A), with macrophages constituting about 40% of these cells (CD45^+^CD11b^+^HLA‐DR^+^ cells, Figure 2B). Manually dividing the global macrophage population according to high and low marker expression for each patient (Figure S2B), we observed that the CCR7 M1‐associated marker was highly expressed in our macrophage population (CD45^+^CD11b^+^HLA‐DR^+^ cells, Figure 2C). In addition to this conventional sequential biaxial plot‐based analysis, we utilized t‐SNE visualization to evaluate macrophages within the total immune cell (CD45^+^ cells) population of each patient. On the global t‐SNE map, 8 main clusters were identified by smooth density plots (Figures 2D and S2A). Of these clusters, cluster #1 (red) and cluster #8 (light green) were myeloid populations expressing both high levels of CD11b and HLA‐DR (Figures 2D and S2A). Interestingly, cluster #8 represents a macrophage population that expresses both M1 (CCR7) and M2 (CD163, CD206, B7‐H3, and PD‐L1) markers (Figure 2D). By combining these conventional and t‐SNE analyses, we observed that CD163^high^ macrophages also expressed high levels of CCR7. The same observations were made with CCR7 and all the other M2‐associated markers, supporting high coexpression of both M1 and M2 markers in human PCa‐associated macrophages (Figure 2E). Moreover, starting from global immune cell population to the macrophage population and then the CCR7^high^ macrophage population on the global t‐SNE analysis, we also detected an enrichment for CD163^high^, CD206^high^, PD‐L1^high^, B7‐H3^high^ expression (Figure S2C).

To corroborate our findings, we analyzed M1 and M2‐marker expression in the TCGA (*n* = 333) and GSE21032 (*n* = 131) gene expression datasets of primary PCa. In both datasets, expression of M1 (*CCR7*) and M2 markers (*CD163*, *CD206*, and *CD274(*) were positively correlated (Figure 2F). Taken together, these data strongly suggest a dual expression of both M1 and M2 markers exists on macrophages present in prostate tumors.

### 3.3 PCa cells subvert M1 macrophages into macrophages with dual M1 and M2 characteristics

**FIGURE 3:**
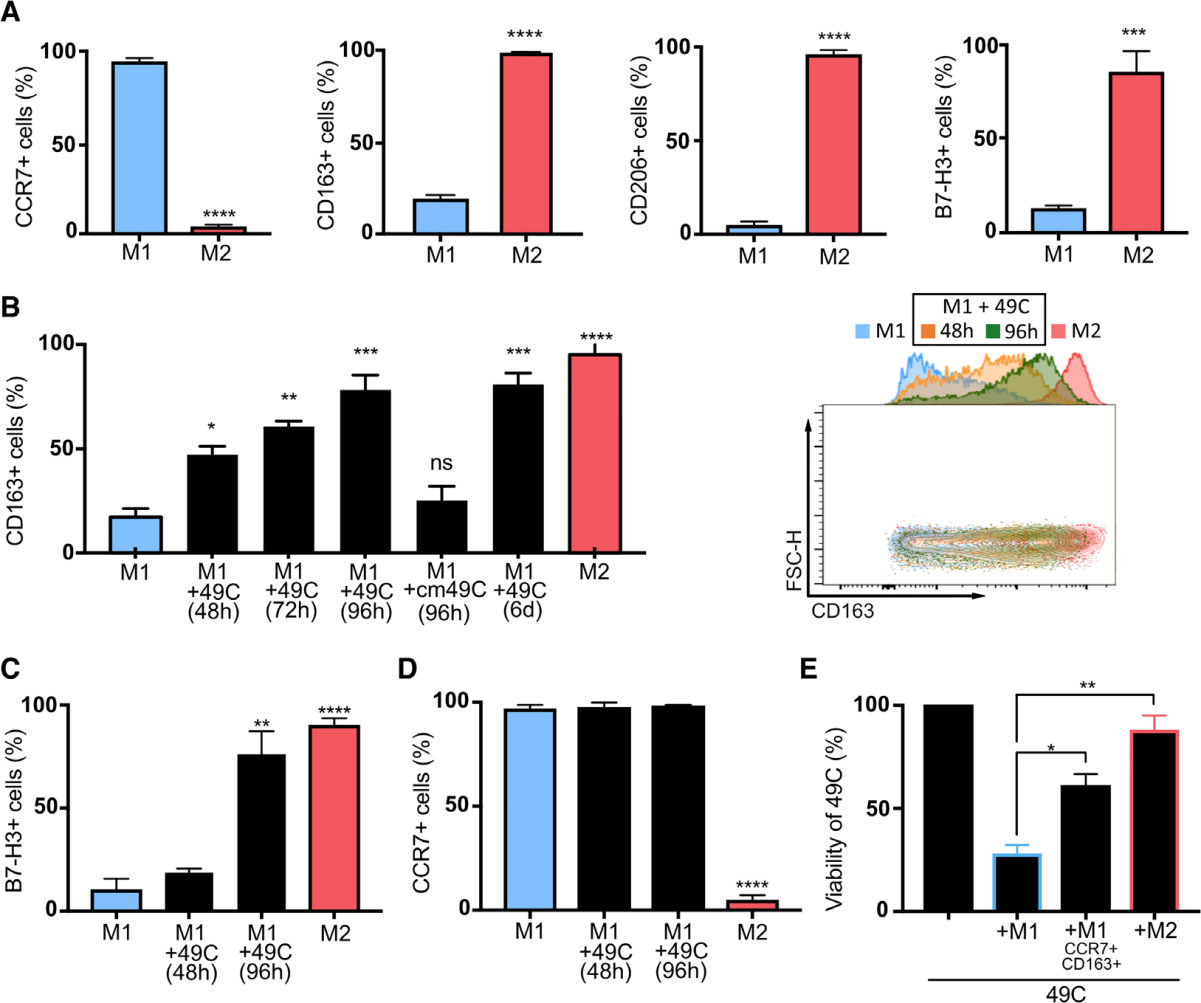
M1 macrophages are subverted by PCa cells with loss of cytotoxic function. Freshly isolated monocytes were polarized into M1 or M2 macrophages during 96 h. (A) Cells were analyzed by flow cytometry using common macrophage markers (CD45, HLA‐DR, CD11b), M1 markers (CCR7), M2 markers (CD163, CD206), and immune checkpoints (B7‐H3). (B)–(D) To evaluate the effect of PCa cells on M1 macrophages, freshly isolated monocytes were polarized into M1 or M2 macrophages during 24 h then cocultured with 49C^ENZR^ cells in 1:1 ratio for 48 h to 6 days (6 days). M1 macrophages were also cultured with 49C^ENZR^ cell conditioned media (cm49C) for 96 h. Phenotype of harvested macrophages was analyzed by flow cytometry and proportion of (B) CD163+ cells, (C) B7‐H3+ cells, and (D) CCR7+ cells are quantified. 49C^ENZR^ cells were cultured alone, with freshly polarized M1 macrophage or with M1 previously cultured with 49C^ENZR^ and sorted to select cells with double expression of CCR7 and CD163 (M1CCR7+CD163+). (E) Cytotoxic activity of macrophages was evaluated by measuring the percentage of viable 49C^ENZR^ (CD45– fraction) within each coculture. Data represent the means ± SEM (*n* = 3). Statistical analysis was performed using Student's *t*‐test, with the levels of significance defined as ^*^ *p* < .05, ^**^ *p* < .01, ^***^ *p* < .001, and ^****^ *p* < .0001

Dual expression of M1 and M2 markers on macrophages infiltrating prostate tissues suggests that the TME reeducates infiltrating inflammatory macrophages toward a mixed phenotype. We therefore sought to explore this hypothesis using MDMs and human PCa cells. First, M1 and M2 macrophages were produced from CD14^+^ monocytes isolated from whole blood of healthy male donors according to validated protocols. As expected, we observed high expression of CCR7 and low expression CD163, CD206, and B7‐H3 in M1 polarized macrophages (Figures 3A and S3A). Further, we observed that M1 macrophages secrete IL‐12 and TNF‐α (Figure S3B) and inhibit the proliferation of PCa cells. To assess direct effects of PCa cells on human macrophages, we used a direct coculture model. Different PCa cells were tested and 49C^ENZR^ cells were selected because of their robustness and increased capacity to induce CD163 expression (Figure S3A, C). Following macrophage M1 polarization, 49C^ENZR^ cells in a 1:1 ratio or 49C^ENZR^ cells conditioned media were added for 48 h to 6 days (Figure 3B). Following contact with these PCa cells, M1 macrophages showed an increased proportion of cells expressing M2 markers, with 60–70% of the cells expressing CD163 and B7‐H3 after 96 h of coculture (Figure 3B, C) and a concurrent increase of marker intensity (Figure S3E). CCR7 intensity at the cell surface decreased twofold but 100% of cells remained positive for this marker (Figures 3D and S3E). The proportion of PD‐L1^+^ macrophages was significantly reduced after 48 or 96 h of coculture with 49C^ENZR^ cells (Figure S3D) while that of CD206^+^ cells was not increased (Figure S3F). Notably, these changes were not observed when macrophages were put in direct coculture with PZ‐HPV7 benign prostate epithelial cells (Figure S3F) or with 49C^ENZR^ cells conditioned media (Figure 3B). We observed the same induction of CD163 when M1 macrophages were cultured with other PCa cell lines (LAPC4, 49C^ENZR^, LNCaP) (Figure S3C).

Next, we sought to evaluate the anticancer properties of dual CCR7^+^ and CD163^+^ macrophages (M1^CCR7+/CD163+^) in our model. Cell sorting by flow cytometry was used to isolate M1^CCR7+/CD163+^ macrophages, which were subsequently put in direct coculture with PCa cells as above. These experiments confirmed that these subverted macrophages significantly lost their cytotoxicity towards 49C^ENZR^ cells compared to M1 macrophages but did not completely resemble M2 macrophages (Figure 3E).

### 3.4 M1 and M2 macrophages can switch their phenotype

**FIGURE 4:**
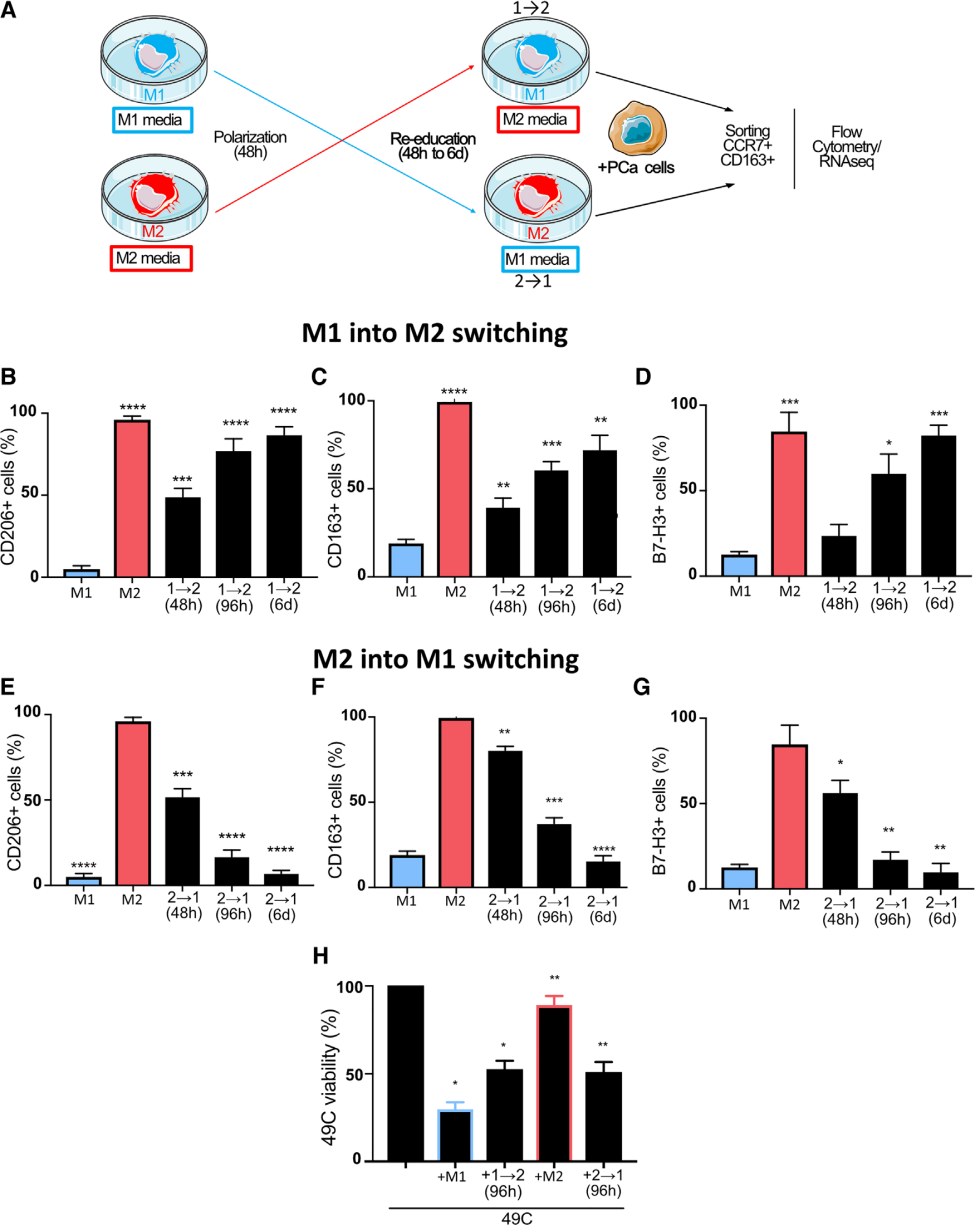
M1 and M2 macrophages can switch their phenotypes to M2 and M1 phenotypes, respectively. (A) Schema of reeducation protocol of macrophages by switching culture media. MDM from healthy donors were polarized into M1 or M2 macrophages for 48 h. After 48 h, cells were washed and conditioned culture medium of M2 macrophages was added onto M1 macrophages (1→2) or vice versa (2→1) for another 48 h to 6 days of culture. Cells were then harvested, stained with antibody cocktail and analyzed by flow cytometry. Proportion of (C) CD206+ cells, (D) CD163+ cells, and (E) B7‐H3+ cells are quantified after M1 into M2 switching. Proportion of (F) CD206+ cells, (G) CD163+ cells, and (H) B7‐H3+ cells are quantified after M2 into M1 switching. 49C^ENZR^ cells were cultured alone, with freshly polarized M1 or M2 macrophages or with 1→2 or 2→1 macrophages. (I) Macrophage population was excluded by CD45 marker and percentage of viable 49C^ENZR^ cells are presented. Data represent the means ± SEM (*n* = 3). Statistical analysis was performed using Student's *t*‐test, with the levels of significance defined as ^*^ *p* < .05, ^**^ *p* < .01, ^***^ *p* < .001, and ^****^ *p* < .0001

Reactivation of the antitumor function of subverted tumor associated macrophages necessitate their repolarization into an inflammatory M1 phenotype. To further characterize the plasticity of macrophages, we next assessed the capacity of polarized macrophages to be reeducated into their opposing phenotypes through exposure to conditioned culture media of polarized macrophages. Freshly isolated CD14^+^ monocytes from healthy donors were polarized into M1 or M2 macrophages as above, followed by an exchange of culture media (e.g., culture medium of M1 macrophages replaced that of M2 macrophages (1→2), and vice‐versa; Figure 4A). We observed that both M1 and M2 phenotypes can be repolarized into the opposing phenotype in a time‐dependent manner. After 4–6 days of repolarization, an increasing proportion of 1→2 macrophages expressed CD163, CD206, and B7‐H3 markers on their surface and after 6 days the proportions were close to that observed in M2 macrophages (Figure 4B–E). The intensity of cell surface expression also increased over time (Figure S4A). Conversely, M2 macrophages reeducated with M1 media showed a decreasing proportion of cells positive for CD163, CD206, and B7‐H3 markers (Figure 4E–G). After 6 days, less than 20% of M2 macrophages reeducated to M1 (2→1) still expressed CD163, CD206, or B7‐H3. We also observed a concordant decrease in the intensity of cell surface expression over time (Figure S4D). Interestingly, CCR7 expression was maintained independently of the direction of reeducation (Figure S4B, E). We also observed that PD‐L1 expression found in nearly 100% of M1 macrophages (Figure S3A, D) was downregulated by the transition from M1 to M2 as very few cells expressed it after the transition, while the proportion of cells expressing PD‐L1 was significantly increased when M2 macrophages were reeducated into M1 macrophages (Figure S4C, F).

### 3.5 Direct interaction of M1 and M2 macrophages with PCa cells prevent their reeducation

**FIGURE 5:**
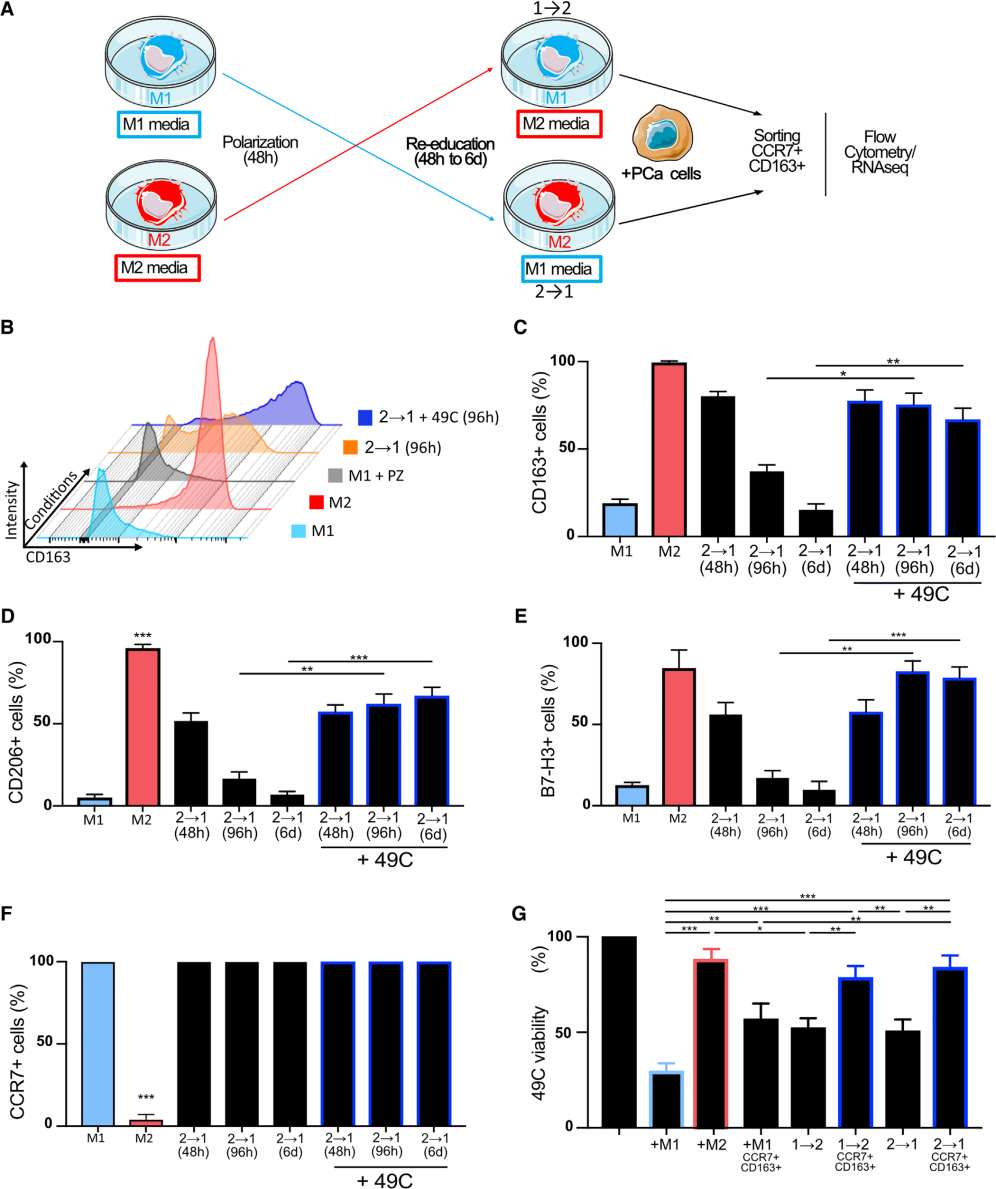
49C^ENZR^ cells inhibit M2 into M1 macrophage reprogramming to favor TAM phenotype. (A) Schema of macrophage reeducation by switching of media in presence of PCa cells. 49C^ENZR^ cells were cultured alone, with freshly polarized M1 or M2 macrophages, with M1 macrophages previously cocultured with 49C^ENZR^ cells and sorted by flow cytometry to select cells with double expression of CCR7 and CD163 (M1 CCR7+ CD163+), with M1 macrophages previously reeducated into M2 (1→2) or the inverse (2→1) or with M1 previously reeducated into M2 and cocultured with 49C^ENZR^ cells then sorted to select cells with double expression of CCR7 and CD163 (1→2 CCR7+ CD163+) or the inverse (2→1 CD163+ CCR7+). After coculture, cells were harvested, stained with antibody cocktail, and analyzed by flow cytometry for marker expression. (B) M1 macrophages were also cultured with epithelial normal prostatic cell line (PZ‐HPV7), PCa cell line (49C^ENZR^ cells) or alone. Proportion of (C) CD163+ cells, (D) CD206+cells, (E) B7‐H3+ cells, and (F) CCR7+ cells are quantified. (G) 49C^ENZR^ cells viability was evaluated on CD45– marked with viability stain after 96 h of coculture. Results are presented as percentages (%). Data represent the means ± SEM (*n* = 3). Statistical analysis was performed using Student's *t*‐test, with the levels of significance defined as ^*^ *p* < .05, ^**^ *p* < .01, ^***^ *p* < .001, and ^****^ *p* < .0001

To understand how reeducation of macrophages would occur in the context of the prostate TME, we performed coculture experiments of polarized macrophages in the presence of 49C^ENZR^ PCa cells (Figure 5A). These experiments demonstrated that the presence of PCa cells promotes the retention of M2‐associated markers during reeducation toward a M1 phenotype (Figure 5). Around 65–85% of M2 macrophages reeducated into M1 macrophages (2→1 macrophages) retained high levels of CD163, CD206 and B7‐H3 expression (Figures 5B–E and S5A). Notably, CCR7 expression was not affected by direct coculture with 49C^ENZR^ cells (Figures 5F and S5A). However, the presence of PCa cells prevented the increase in the proportion of PD‐L1^+^ in 2→1 macrophages (Figure S5F). On the other hand, the presence of 49C^ENZR^ cells appear to facilitate the switching of M1 to M2 (1→2) macrophages since we observed a greater proportion of macrophages expressing CD163, CD206, and B7‐H3 and a higher number of respective cell surface molecules when cultured in presence of PCa cells (Figure S5B–D).

As above, we used cell sorting by flow cytometry to isolate dual positive CCR7^+^ CD163^+^ macrophages obtained from these reeducation experiments in presence of 49C^ENZR^ cells (1→2^CCR7+/CD163+^ or the inverse 2→1^CCR7+/CD163+^) in order to assess their cytolytic function. We observed that subverted macrophages (1→2^CCR7+/CD163+^ and 2→1^CCR7+/CD163+^) exert similar protumoral function favoring PCa cell viability compared to PCa cells cocultured with M1 (Figure 5G). M1 macrophages reeducated into M2 macrophages in the presence of PCa cells had a less antiproliferative activity compared to 1→2 reeducation without PCa cells (1→2^CCR7+/CD163+^ vs. 1→2, Figure 5G). Similarly, the presence of PCa cells during 2→1 reeducation (2→1^CCR7+/CD163+^ vs. 2→1) resulted in a loss of M1 macrophage antiproliferative activity (Figure 5G). Reeducation of both macrophage groups in the presence of PCa cells was accompanied by a significant decrease in PD‐L1 expression (Figure S5E, F).

### 3.6 Macrophage reeducation in presence of PCa cells leads to concomitant specific changes in chemokine signature for M1^CCR7+/CD163+^ and 2→1^CCR7+/CD163+^

**FIGURE 6:**
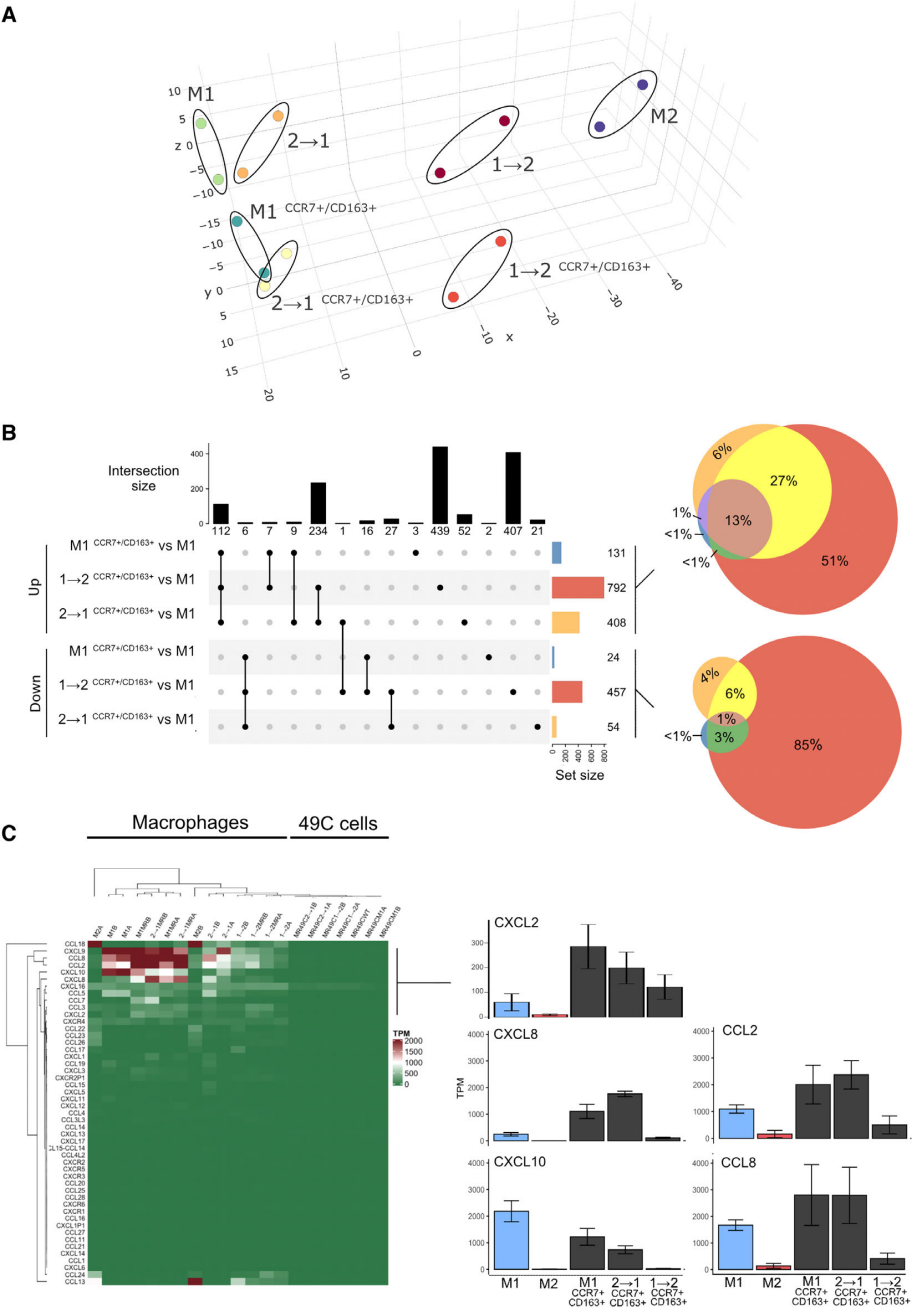
49C^ENZR^ cells induce macrophage reprogramming of chemokine network during reeducation. Principal component analysis based on the top 500 genes with the higher variance across the transcriptome expressed during macrophage polarization and switch of phenotypes and subversion by PCa cells. (A) Sample trend during polarization and re education is shown in a scatter plot of the principal components 1 and 2, which summarize 98% of the system variance. Two donors (A and B) were studied. (B) Upset plot representing intersection size and set size between each compared group of up‐ and downregulated genes within each group or subverted macrophages using M1 as reference. (B, right) Venn diagram of up‐ and downregulated genes within each group or subverted macrophages using M1 as reference. (C) Heatmap of the count matrix based on the chemokine (CXCL‐ and CCL‐motif) genes with the highest number of counts across the samples. (D) Bar graphs of the transcripts per kilobase million (TPM) of CXCL2, CXCL8, CXCL10, CCL2, and CCL8 in each sample

We next performed RNA‐seq to evaluate the gene expression similarities and differences of macrophages reeducated by the cytokine milieu compared to the changes induced by direct coculture with 49C^ENZR^ PCa cells (Figure 6). Peripheral blood mononuclear cells (PBMCs) from two healthy male donors were used to derive concurrently all experimental samples. This included total macrophages (M1, M2, 1→2, or 2→1) and flow‐sorted macrophages from mixed cocultures (M1^CCR7+/CD163+^, 1→2^CCR7+/CD163+^, or 2→1^CCR7+/CD163+^). Principal component analysis demonstrated overall highly comparable results for biological replicates and overall differences in transcriptomic profiles (Figure 6A). Macrophages polarized by media conditions (M1, M2, 1→2, 2→1) clustered separately from PCa‐educated macrophages (M1^CCR7+/CD163+^, 1→2^CCR7+/CD163+^, or 2→1^CCR7+/CD163+^). As expected, differential expression analysis revealed more differentially expressed genes between M1 versus M2 macrophages (1006 upregulated and 784 downregulated; false discovery rate [FDR] < 5%) compared to reeducated macrophages 1→2 and M2 (350 upregulated and 169 downregulated; FDR < 5%) or 2→1 and M1 (12 upregulated and 8 downregulated; FDR < 5%, Figure 6B, Table S4). Macrophages cocultured with 49C^ENZR^ cells (M1^CCR7+/CD163+^, 1→2^CCR7+/CD163+^, or 2→1^CCR7+/CD163+^) had relatively more upregulated genes compared to downregulated genes (Table S4). We observed that mRNAs from recognized PCa‐associated genes (e.g., *AR*, *KLK3*, *STEAP2*, *NKX3.1*) were very high in the list of differentially upregulated macrophage genes isolated from cocultures relative to macrophage controls (Supplementary Data), suggesting significant phagocytosis of mRNA from PCa cells. Therefore, instead of global pathway analyses we focused on the chemokine network of these subverted macrophages, which we observed were exclusively expressed in macrophages (Figure 6C). M1 was selected as a reference to decipher how the M1 inflammatory function of infiltrating macrophages could be subverted. We found that both M1^CCR7+/CD163+^ and 2→1^CCR7+/CD163+^ macrophages shared similar chemokine alterations, with higher transcripts for chemokines *CXCL2*, *CXCL8*, *CCL2*, and *CCL8*, but fewer *CXCL10* transcripts (Figure 6C, D). These changes were most pronounced for *CCL2*, *CCL8* and *CXCL2* among 2→1^CCR7+/CD163+^ macrophages. Together, these results suggest that the PCa‐induced changes in TAMs may result in abnormally high levels of production of certain chemokines implicated in myeloid cell recruitment.

## 4 DISCUSSION

As an abundant immune component in prostate tumors, there is interest whether strategies to target prostate TAMs may be an effective immunotherapeutic approach for PCa.^24^ Our study highlights the importance of the plasticity of macrophages within the prostate TME. Our investigation of our model of prostate TAMs elucidates the relative contributions of the cytokine milieu and PCa cell contact on subverting infiltrating inflammatory macrophages to become TAMs. Our patient and in vitro results showing the persistence of inflammatory markers suggest that prostate TAMs originate mainly as infiltrating myeloid cells, which are then subverted by prostate tumor cells. The potent protumoral changes, which are induced in human inflammatory macrophages through contact with PCa cells implies that strategies to repolarize these macrophages must concomitantly destroy the tumor cells to be sufficiently effective. Together, our study adds important information to prior studies, which associate immunosuppressive macrophages with more aggressive, poor prognosis PCa.^20^, ^25^

The finding that CD163^+^ cells in tumor adjacent normal epithelia was more prognostic than CD163^+^ cells within the tumor core was unexpected and striking in magnitude. At first glance, this appears in contrast to our findings of the strong immunosubversive potency of direct PCa cell contact with macrophages relative to diffusible factors. However, in patient tumors, we observed that CD163 expression was always higher in tumoral regions; it was the relative increased density in normal adjacent areas, which was independently predictive of long‐term risk of metastasis and PCa‐related death. One possibility suggested by our studies to explain these findings may be that the dual nature of tumor‐subverted macrophages facilitates their exfiltration to adjacent normal tissue. The presence of CD163^+^ macrophages in this adjacent prostate epithelium may be a feature of broader immunosuppression of normal antitumor inflammation, which arises over time and with treatment (e.g., following androgen deprivation therapy).^26^

Our multiparametric flow cytometry analyses, while spatially limited, provide important detail beyond immunohistochemistry about prostate TAMs. We identified a relative abundance of macrophages, which express both M1 and M2 phenotypic characteristics. CD163 and CD206 are known prostate TAM markers,^27^ though a role for CCR7^+^ macrophages in PCa has not been reported to our knowledge. Expression of B7‐H3, associated with poor prognosis PCa, appears to be altered on macrophages in a similar manner to other M2 markers.^5^, ^28^ Based on our data, we propose that M1 macrophages recruited into the TME of PCa are subverted by PCa cells and the surrounding immunosuppressive milieu to become TAMs with protumoral functions, low expression of PD‐L1 and high CD163, CD206, and B7‐H3 expression. The low expression of PD‐L1 in both prostate TAMs and our MDM‐derived model was unexpected given its understood role in immunosuppression but highlights the importance of tissue‐specific evaluation. Notably, the expression of PD‐L1 in human prostate cancer has previously been reported to be low in normal adjacent prostate tissue tumor‐infiltrating lymphocytes/macrophages.^29^

While at the same time demonstrating the plasticity of human macrophages, our model suggests that in the presence of PCa cells, human macrophages undergo certain changes, which cannot easily be surmounted. We observed that in the presence of PCa cells, an M1 phenotype could not be fully restored even with M1‐inducing cytokine stimulation. In addition to losing their capacity to revert to the original M1 inflammatory phenotype, we demonstrate functional differences with diminished cytotoxicity. This has implications for PCa immunotherapy; to date immune checkpoint, inhibition remains effective only for a very small proportion of advanced PCa patients.^30^ Our results suggest that attempts to repolarize prostate TAMs will not be sufficiently effective without concomitantly eradicating adjacent tumor cells. Results of the ongoing KEYNOTE‐921 and CheckMate‐7DX Phase III trials evaluating combination docetaxel with PD‐1 inhibition in metastatic CRPC may provide further clinical validation of this concept. Further, our RNA‐sequencing data suggest that in the presence of PCa cells, an overproduction of certain chemokines is induced in macrophages. These induced chemokines may contribute to the very high levels of myeloid cells present in advanced prostate tumors.^31^ This model suggests potential mechanisms how TAMs promote PCa progression and presents a platform for further mechanistic studies.

There are some limitations to our study. CD163^+^ macrophages on immunohistochemistry were identified based on morphology without multiplex characterization. Similarly, our gating strategy using CD45, CD11b, and HLA‐DR may include dendritic cells, though in our experience these are relatively rare in the prostate. Similarly, CD163 may identify both monocyte and macrophage cells, though this unlikely to affect our results as monocytes differentiate once in prostate tissue. Only 21 patients underwent the identical full panel of immunosuppressive markers for our flow cytometry studies, with almost all these patients having GGG ≥3 disease on final pathology. Our selection for more aggressive, relatively focal disease may incur unknown bias into our results. In our coculture model, the phagocytic activity of macrophages limited our capacity to accurately evaluate the proliferation of PCa cells using CSFE (data not shown).^32^ This phagocytic activity also may have influenced the RNA sequencing results, though a recent single‐cell analysis of human lymph nodes suggests our findings of macrophage *KLK3* expression may reflect adverse PCa biology in patients.^33^ While our RNA‐sequencing showed no detectable chemokine expression, this is reported in other PCa cell lines.^34^ Further, while our model appears reproducible between different healthy male volunteers, there remains the possibility significant interpersonal variation may alter the results. Our studies highlight that our human prostate TAM model mirrors the phenotype observed in human prostate tumors, though most of our experiments were performed with one PCa cell line. Further studies evaluating other cancer cell lines, particularly those associated with different TAM phenotypes (e.g., in colon, gastric cancers) will provide further insights into interactions induced by these infiltrating myeloid cells.

In summary, we demonstrate how PCa may subvert infiltrating inflammatory macrophages to have a dual immunosuppressive and inflammatory phenotype. Our results indicate the presence of such subverted dual inflammatory and immunosuppressive macrophages in normal prostate tissue is a characteristic of poor‐prognosis tumors. Further, our results evaluating the plasticity of TAMs suggest targeting prostate TAMs without concomitant eradication of PCa cells may be ineffective. Additional research is needed to identify feasible and effective strategies for therapeutically targeting the immunosuppressive prostate TME.

## CONFLICT OF INTEREST

FP reports research funding from Astellas, Janssen, Bayer as well as personal fees as a consultant for Amgen, Bayer, Sanofi, Astellas, Astra Zeneca, Tersera and Janssen. AB reports research funding from Astellas, IMV Inc, GSK biologicals as well as personal fees as a consultant from Merck. YF reports research funding from Tersera, Astellas, IMV inc, as well as personal fees as a consultant from Merck, Sanofi, Ferring, Amgen, Janssen, Astellas. PT reports research funding from Bristol‐Myers‐Squibb, Sanofi and Janssen as well as personal fees as a consultant from Bayer, Ferring, TerSera, Janssen, Sanofi, and Abbvie. The other authors have no conflicts of interest to declare.

## Supporting information

Supporting information
**Figure S1**. CD163+ cell infiltration pattern in tumor and tumor‐adjacent normal epithelium
**Figure S2**. High‐dimensional t‐SNE visualization and manual gating strategy of patient cancerous prostate flow cytometry analyses
**Figure S3**. PCa cells reprogram M1 macrophages into M2 macrophages expressing CD163, CD206, and B7‐H3
**Figure S4**.  M1 and M2 macrophages can be reprogrammed into respective M2 and M1 phenotypes
**Figure S5**. 49C cells favor M2‐like macrophage reprogramming

Supporting information
**Table S1**. List of antibodies used in multiparametric flow cytometry analyses
**Table S2**. Clinicopathologic characteristics of the cohort used for CD163 immunohistochemistry staining
**Table S3**. Cox regression analyses for CD163 expression as predictor of long‐term clinical outcomes
**Table S4**. Number of differentially expressed genes between macrophage conditions
**Data file S1**. Differentially expressed genes (DEG) among different monocyte‐derived macrophage conditions
