# Supplementary material for: Subversion of infiltrating prostate macrophages to a mixed immunosuppressive tumor‐associated macrophage phenotype
Source: Clin Transl Med. 2022 Jan 24;12(1):e581. doi: 10.1002/ctm2.581 (PMC8786699; doi:10.1002/ctm2.581)
Supplement: Supplementary file 1 — Supporting information Figure S1. CD163+ cell infiltration pattern in tumor and tumor‐adjacent normal epithelium Figure S2. High‐dimensional t‐SNE visualization and manual gating strategy of patient cancerous prostate flow cytometry analyses Figure S3. PCa cells reprogram M1 macrophages into M2 macrophages expressing CD163, CD206, and B7‐H3 Figure S4. M1 and M2 macrophages can be reprogrammed into respective M2 and M1 phenotypes Figure S5. 49C cells favor M2‐like macrophage reprogramming [file CTM2-12-e581-s001.pdf]

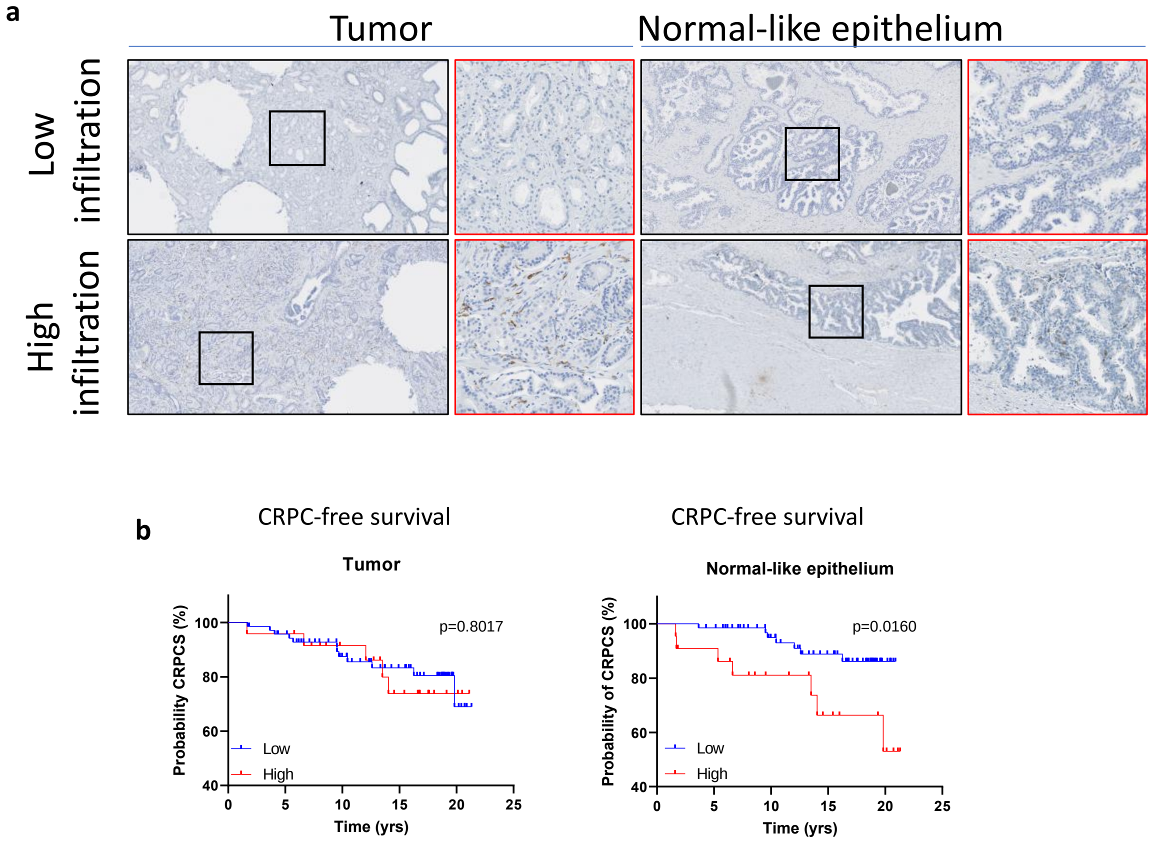

Figure S1: CD163^{+} cell Infiltration pattern in tumor and tumor-adjacent normal epithelium. (a) Examples of low and high infiltration in both areas. Panels with red borders correspond to enlargement of areas delineated by squares with black borders in the left panels. (b) Kaplan-Meier curves showing the castrate-resistant prostate cancer(CRPC)-free survival according to the level of CD163^{+} cell infiltration (4th quartile/high (red line) vs 1st-3rd quartiles/low (blue line)) in the tumor and in the tumor-adjacent normal-like epithelium areas are presented. Log-rank test was used to assess the significance of the differences observed.

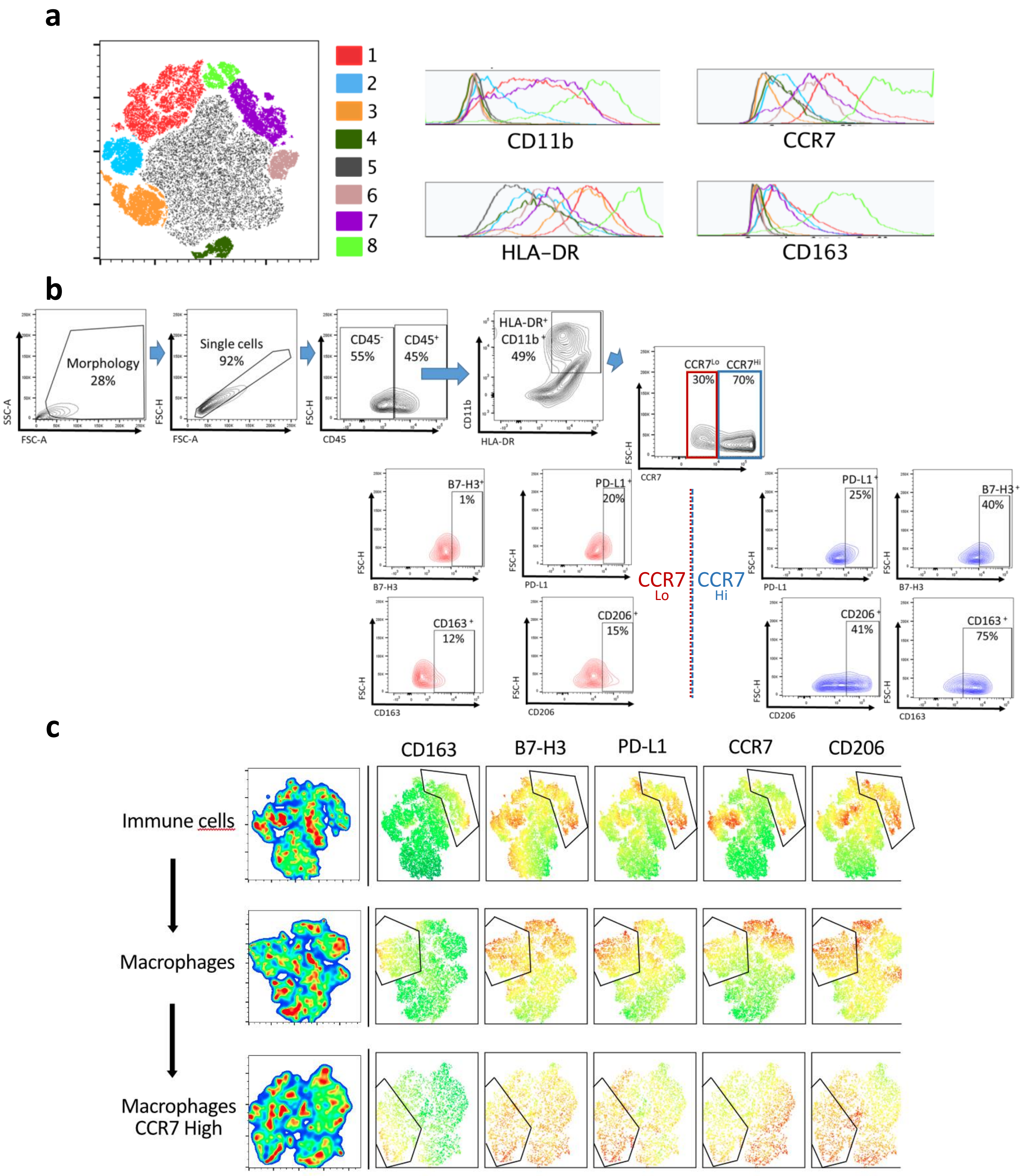

Figure S2: High dimensional t-SNE visualization and manual gating strategy of patient cancerous prostate flow cytometry analyses. t-SNE-guided manual gating analysis of immune population ($CD45^+$ cells) of 21 pooled patients of the cohort with the signal strength of key phenotypic markers (HLA-DR, CD11b, CCR7 and CD163) defining specific cellular lineages expressed with a blue-green-yellow-red continuous color scale. (a) Macrophage population (light green) was delineated by high expression of the 4 phenotypic markers on the t-SNE map and by histogram evaluation. (b) Example of macrophage manual gating strategy for prostate biopsy sample. (c) Example of individual patient gating for total immune cell population ($CD45^+$ cells), total macrophage population ($CD45^+$ $CD11b^+$ $HLA\text{-}DR^+$ cells) and CCR7 high macrophage population ($CD45^+$ $CD11b^+$ $HLA\text{-}DR^+$ $CCR7^{Hi}$ cells) on t-SNE analysis.

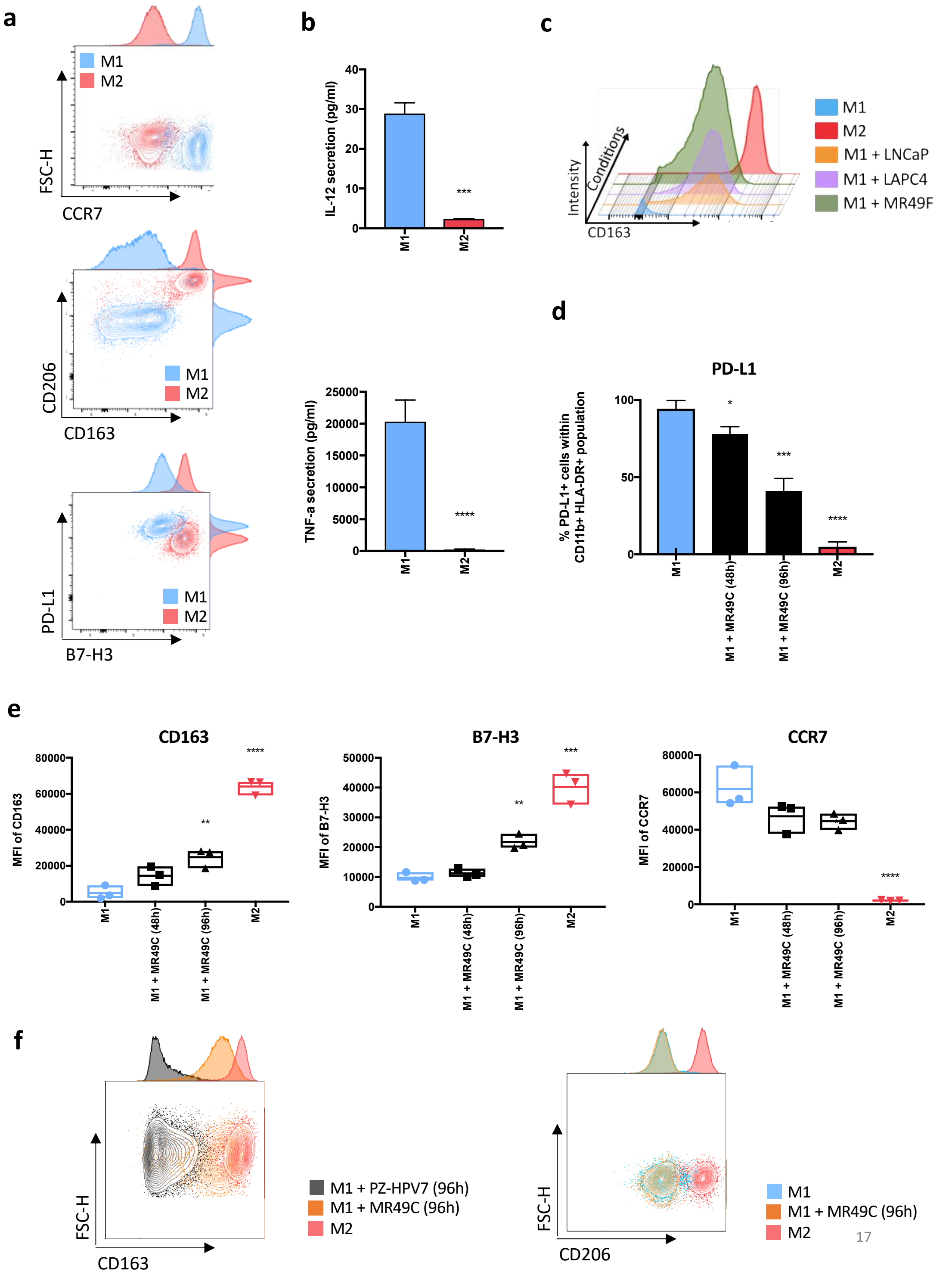

Figure S3: PCa cells reprogram M1 macrophages into M2 macrophages expressing CD163, CD206 and B7-H3. Monocyte-derived macrophages were polarized into M1 and M2 macrophages over 96h. (a) Spectral views of both populations showing expression of CCR7, CD163, CD206, B7-H3 and PD-L1. (b) Supernatants of M1 and M2 macrophages were tested by bioplex assay for presence of IL-2 and TNF-α. (c) M1 macrophages polarized for 24h were co-cultured with PCa cell lines LNCaP, $49F^{ENZR}$ or LAPC4 in a 1:1 ratio for an additional 96h. Effect of the co-culture on CD163 expression on macrophages was evaluated by flow cytometry. (d) M1 macrophages polarized for 24h were co-cultured with 49C cells in 1:1 ratio during 48 or 96h. Then PD-L1 expression was analyzed by flow cytometry. (e) MFI was calculated within the macrophage ($CD45^{+}$ $HLA\text{-}DR^{+}$ $CD11b^{+}$) population using FlowJo. (f) After 24h of polarization, M1 macrophages were co-cultured with $49C^{ENZR}$ cells or PZ-HPV7 cells (benign prostatic epithelial cells) for 96h and evaluated by flow cytometry. Co-culture experiments and marker analyses were controlled with monocultures of M1 and M2 macrophages. Data represent the mean ± SEM (n=3). A student t-test was used to compare means, with the levels of significance defined as *p<0.05, **p<0.01, ***p<0.001 and ****p<0.0001.

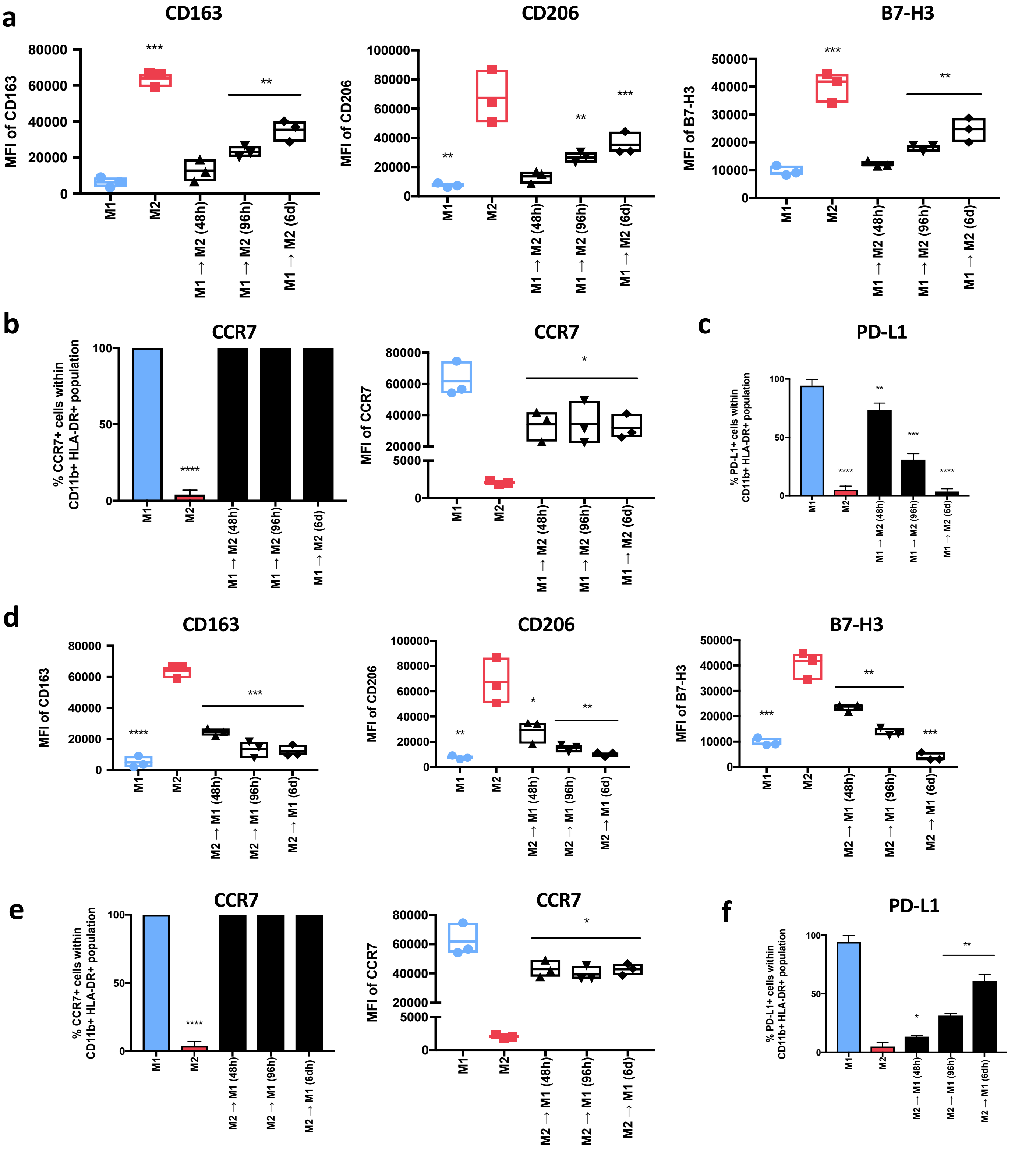

Figure S4: M1 and M2 macrophages can be reprogrammed into respective M2 and M1 subtypes. MDM were polarized into M1 or M2 macrophages for 48h. After 48h, cells were washed and conditioned culture media of M1 macrophages was added to M2 macrophages and vice versa for another 48h to 6 days. (a) Increases in M2 median fluorescence intensity (MFI) were seen as M1 macrophages were re-educated with M2 media. (b) However, CCR7 expression remained persistently high and PD-L1 expression decrease during the same media re-education (c) Conversely, re-education of M2 macrophages with M1 media decreased M2 markers. (e) Re-education of M2 macrophages with M1 media rapidly increased CCR7 expression. (f) A partial increase in PD-L1 expression was induced during the same media re-education. For each marker, MFI was calculated within the $CD45^{+}$ HLA-DR^{+} $CD11b^{+}$ macrophage population with FlowJo software. CCR7 expression was evaluated by flow cytometry analysis during macrophage reprogramming by gating on $CD45^{+}$ HLA-DR^{+} $CD11b^{+}$ (c and f). Data represent the mean±SEM (n = 3). A student t-test was used to compare means, with the levels of significance defined as *p<0.05, **p<0.01, ***p<0.001 and ****p<0.0001.

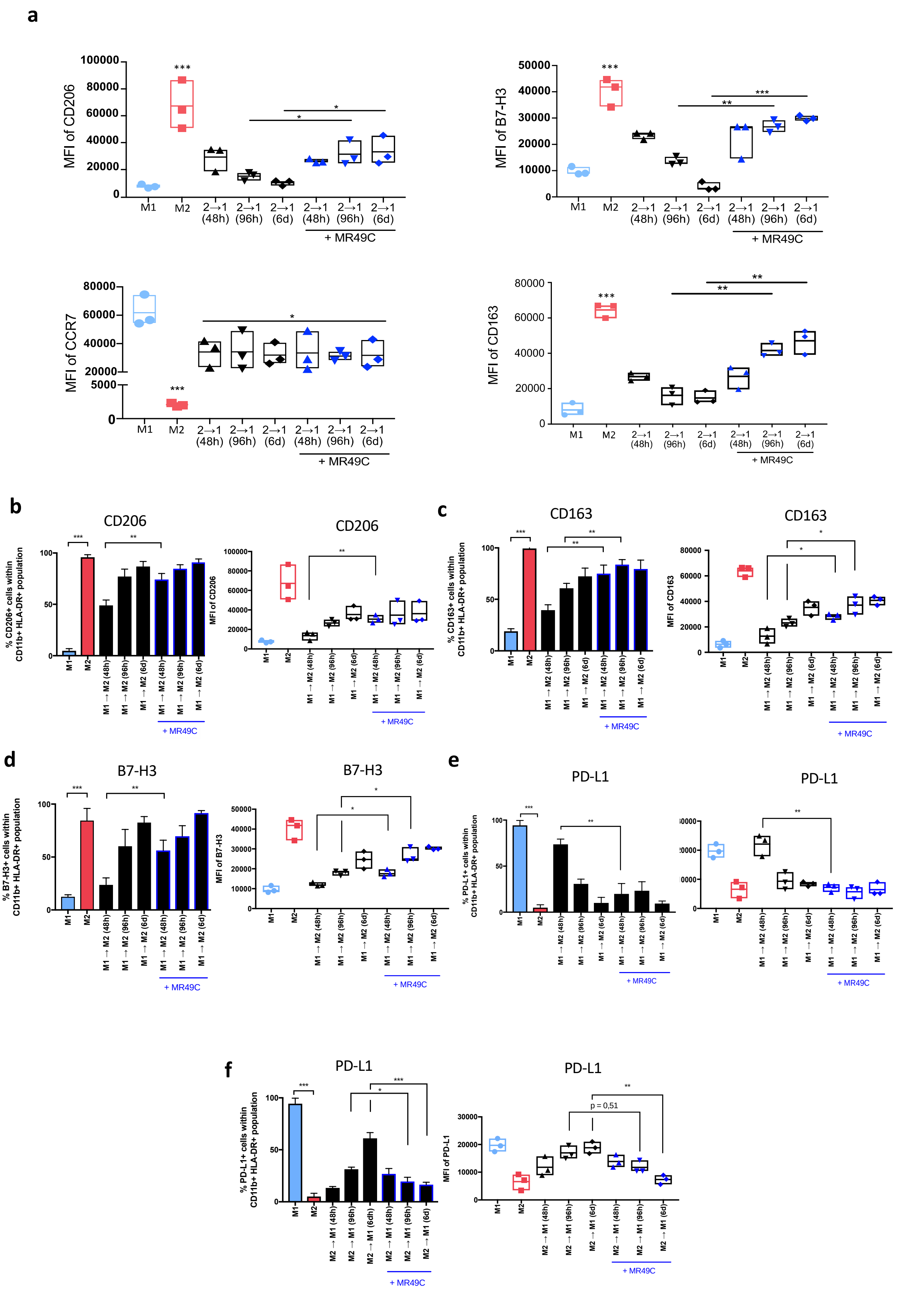

Figure S5: 49C^{ENZR} cells favor M2-like macrophage reprogramming. Monocyte-derived macrophages from healthy donors were polarized into M1 or M2 macrophages for 48h. After 48h, cells were washed and conditioned M1 media was added into M2 macrophages or M2 media was added into M1 macrophages with 49C^{ENZR} cells for another 48h to 6 days of culture then cells were harvested, and multi-parametric flow cytometry analysis of the markers was performed. (a) The presence of 49C^{ENZR} cells abrogated the decrease in CD163 ,B7-H3 and CD206 induced by M1 media, but had no effect of CCR7. (b-e) The presence of 49C^{ENZR} cells had minimal or slight increase on CD206, CD163 and B7-H3 induced by M2 media, and increased reduction in PD-L1 by M2 media. (f) The increase in PD-L1 by M1 media re-education was decreased by the presence of 49C^{ENZR} cells. Data represent the mean±SEM (n = 3). Statistical analysis was performed using Student's t-test, with the levels of significance defined as *p<0.05, **p<0.01, ***p<0.001 and ****p<0.0001
