# Supplementary material for: Subversion of infiltrating prostate macrophages to a mixed immunosuppressive tumor‐associated macrophage phenotype
Source: Clin Transl Med. 2022 Jan 24;12(1):e581. doi: 10.1002/ctm2.581 (PMC8786699; doi:10.1002/ctm2.581)
Supplement: Supplementary file 2 — Supporting information Table S1. List of antibodies used in multiparametric flow cytometry analyses Table S2. Clinicopathologic characteristics of the cohort used for CD163 immunohistochemistry staining Table S3. Cox regression analyses for CD163 expression as predictor of long‐term clinical outcomes Table S4. Number of differentially expressed genes between macrophage conditions Data file S1. Differentially expressed genes (DEG) among different monocyte‐derived macrophage conditions [file CTM2-12-e581-s002.docx]

**Table S1. Information on antibodies used in multi-parametric flow cytometry analyses.**

| **Reactivity** | **Abs** | **Fluorochrome** | **Clone** | **Isotype** | **#Product** | **Provider** |
| --- | --- | --- | --- | --- | --- | --- |
| Human | CD11b | APC/Cy7 | ICRF44 | Mouse IgG1, κ | 301342 | Biolegend |
| Human | HLA-DR | PerCP-Cy 5.5 | G46-6 | Mouse IgG2a, κ | 552764 | BD Biosciences |
| Human | CD197 | PE | 3D12 | Mouse IgG2a, κ | 552176 | BD Biosciences |
| Human | CD163 | AF647 | GHI/61 | Mouse IgG1, κ | 333620 | Biolegend |
| Human | CD206 | BV605 | 19,2 | Mouse IgG1, κ | 740417 | BD Biosciences |
| Human | B7-H3 | PE/Cy-7 | MIH42 | Mouse IgG1, κ | 351007 | Biolegend |
| Human | PD-L1 | BB515 | MIH1 | Mouse IgG1, κ | 564554 | BD Biosciences |
| Human | PD-L2 | BV421 | MIH18 | Mouse IgG1, κ | 563842 | BD Biosciences |
| Human | PD-1 | PE-CF594 | EH12.1 | Mouse IgG1, κ | 565024 | BD Biosciences |
| Human | CD45 | V500 | HI30 | Mouse IgG1, κ | 560779 | BD Biosciences |
| Mammalian | FVS-780 | APC-Cy7 |  |  | 565388 | BD Biosciences |

**Table S2. Clinicopathologic characteristics of the cohort used for the CD163 IHC staining.**

| **Characteristics** | **n (%)** |
| --- | --- |
|  |  |
| **PSA at diagnosis (ng/ml)** |  |
| ≤10 | 53 (55.8%) |
| 10-20 | 27 (28.4%) |
| >20 | 15 (15.8%) |
| **Gleason (prostatectomy)** |  |
| ≤6 | 22 (23.1%) |
| 3+4 | 33 (34.7%) |
| 4+3 | 17 (17.9%) |
| ≥8 | 23 (24.2%) |
| **T stage (prostatectomy)** |  |
| pT2 (margin +) | 33 (34.7%) |
| pT3-pT4 | 62 (65.2%) |
| **Nodal invasion** | 27 (28.4%) |
| **Surgical Margin** | 76 (80.0%) |
| **Definitive hormone therapy** | 20 (21.0%) |
| **CRPC** | 17 (17.9%) |
| **Biochemical recurrence** | 51 (53.7%) |
| **Metastasis** | 17 (17.9%) |
| **Death** |  |
| Prostate cancer | 13 (13.7%) |
| Other causes | 31 (32.6%) |

**Table S3: Cox regression analyses.** Univariate and multivariate Cox regression analyses for metastasis and prostate cancer(PCa)specific death according to infiltration by CD163^+^ macrophages in the tumor area (CD163_T_) and in the tumor-adjacent normal epithelium (CD163_N_). Multivariate analyses were adjusted for age, PSA, T stage, N Stage, Gleason score and surgical margin status.

|  |  | **Univariate** | | **Multivariate*** | |
| --- | --- | --- | --- | --- | --- |
| **Marker _localisation_** | **Outcome** | **HR (95% CI)** | **p-value** | **HR (95% CI)** | **p-value** |
| **CD163_T_** | CRPC | 1.13 (0.39-3.21) | 0.81 | 0.59 (0.16-2.15) | 0.42 |
|  | Metastasis | 1.11 (0.42-3.40) | 0.73 | 0.44 (0.11-1.73) | 0.24 |
|  | PCa-specific death | 0.80 (0.22-2.92) | 0.73 | 1.06 (0.52-2.16) | 0.87 |
| **CD163_N_** | CRPC | 3.47 (1.20-10.00) | 0.02 | 4.88 (0.97-24.39) | 0.05 |
|  | Metastasis | 5.26 (1.81-15.15) | 0.002 | 9.43 (1.52-58.82) | 0.02 |
|  | PCa-specific death | 5.08 (1.42-18.18) | 0.01 | 3.03 (1.28-7.14) | 0.01 |

**Table S4: Number of differentially expressed genes between macrophage conditions.** Counts were calculated with DESeq2 using a false discovery rate of 5% and a minimum log2 fold change of 1.5. CCR7+/CD163+ in superscript represent macrophages isolated by flow cytometry sorting for both respective antigens.

| Sample | Reference | Number of upregulated  genes | Number of downregulated genes |
| --- | --- | --- | --- |
| M1 | M2 | 1006 | 784 |
| M1^CCR7+/CD163+^ | M1 | 131 | 24 |
| M1^CCR7+/CD163+^ | M2 | 1089 | 722 |
| 1to2^CCR7+/CD163+^ | M1 | 1000 | 749 |
| 1to2^CCR7+/CD163+^ | M2 | 792 | 457 |
| 2to1^CCR7+/CD163+^ | M1 | 408 | 54 |
| 2to1^CCR7+/CD163+^ | M2 | 1267 | 679 |
| 2to1 | M1 | 12 | 8 |
| 1to2 | M2 | 350 | 169 |
| 1to2 | 2to1^CCR7+/CD163+^ | 42 | 364 |
| 2to1 | 1to2^CCR7+/CD163+^ | 42 | 363 |
| M1 | 1to2 | 328 | 289 |
